# Why are there no platypuses at the Olympics?: A teleological case for athletes with disorders of sexual development to compete within their sex category

**DOI:** 10.17159/2078-516X/2020/v32i1a7918

**Published:** 2020-01-01

**Authors:** N Gamble, M Pruski

**Affiliations:** 1 University of Alberta, Faculty of Medicine and Dentistry, Canada; 2 London School of Tropical Medicine and Hygiene, University of London, United Kingdom; 3 Critical Care Science Team, Oxford Road Site, Manchester University NHS Foundation Trust, United Kingdom; 4 School of Healthcare Science, Faculty of Science and Engineering, Manchester Metropolitan University, United Kingdom

**Keywords:** disorders of sexual differentiation, gender, sports performance, innate advantage

## Abstract

In mid-2019, the controversy regarding South African runner Caster Semenya’s eligibility to participate in competitions against other female runners culminated in a Court of Arbitration for Sport judgement. Semenya possessed high endogenous testosterone levels (arguably a performance advantage), secondary to a disorder of sexual development. In this commentary, Aristotelean teleology is used to defend the existence of ‘male’ and ‘female’ as discrete categories. It is argued that once the athlete’s sex is established, they should be allowed to compete in the category of their sex without obligatory medical treatment. Indeed, other athletes who possess advantageous genetic or phenotypic traits that fall outside of the human norm have been allowed to compete as humans without restraint. In both cases, if an athlete possesses the essential attributes of being a human or being male or female they should be permitted to compete in those respective categories; athletes’ eligibilities should not be based upon accidental attributes.

The Court of Arbitration for Sport (CAS) in May 2019 upheld the International Association of Athletics Federation’s (IAAF) regulation for testosterone testing of athletes with disorders of sexual development (DSD), a ruling later supported by the findings of the Swiss Supreme Court.^[1]^ As a result, a prominent female athlete, Caster Semenya, will not be allowed to compete unless her testosterone level is brought within typical female levels. The IAAF acknowledged an element of discrimination but held that this was a ‘necessary, reasonable and proportionate means of achieving the legitimate objective of ensuring fair competition in female athletics in certain events.’ Similarly, the Swiss Supreme Court found that Semenya’s DSD ‘has a direct impact on performance in sport, which could never be achieved by other women,’ ^[2]^ despite the lack of extensive studies and the uncertainty of whether elevated endogenous testosterone levels actually do give an unfair competitive advantage in DSD women.^[3]^ This paper will not engage with the specifics of the CAS’s ruling or the IAAF’s policy, but will rather employ a teleological construct to examine the legitimacy of a female athlete with DSD competing as a female. Similarly, the paper does not address the question of transgenderism in sports, though there certainly may be instances of athletes with DSD who also identify as transgender. These authors are solely concerned with biological sex and not sociological gender.

## Discussion

Firstly, a conspicuous premise needs justification: the existence of only two sexes. Every human organ is oriented towards specific purposes: a heart for pumping blood, eyes for seeing, and lungs for breathing. The sexual arrangement of the human body also has a biological purpose; it is oriented towards human reproduction. However, unlike other human organ systems (e.g. the cardiovascular system), one individual possesses only half of the necessary structures to complete the reproductive system’s function. A female and a male must come together to complete the sexual apparatus. Thus a female is one whose body is oriented towards physiological motherhood, while a male is one whose body is oriented towards physiological fatherhood. These definitions are not simplistically reduced to the difference between male and female gonads, producing large and small gametes, respectively, but also by the males and females distinct arrangements to conceive, protect and nourish children. Humans cannot reproduce on their own, nor does reproduction require three (or more) unique participants. A species where an individual could reproduce on its own, e.g. earthworm, can be considered to have one sex. If a species evolved which featured the necessary reproductive components divided into three discrete units (with members of this species each possessing one of these units), that species would have three sexes.

Human sexual organisation, though, is not reducible to gonads, because if gonads are not present in an individual, one can still discern the purpose of that person’s sexual organisation. Analogously, manned rockets are designed for spaceflight. By looking at the rocket’s design one can perceive this purpose – even if the rocket’s engines are removed or were never installed. If a car’s engine was bolted onto the rocket, it would still be a rocket and not a car. The rocket may not be capable of spaceflight without properly functioning engines, but its purpose is still space travel, and it is still a rocket. Similarly, a person’s sex is an inherent part of that person, even if the gonads are defective or absent – although gonads remain the fundamental means of achieving the purpose of the reproductive system (like the engines of a rocket).

Some contend that those with DSD belong to a spectrum between male and female.^[4]^ However, owing to the nature of human reproduction, there is no meaningful category that can exist between male and female. ^[5]^ Moreover all human foetuses begin on a default path to become female, a path that is only diverted if a sufficient biochemical cascade is both transmitted and received properly within the foetus. DSD occurs when this signal is inappropriately transmitted or received (e.g. an XY foetus with complete androgen insensitivity (CAIS), will develop as a female). In DSD cases, discerning whether a baby is male or female can be difficult, but this merely highlights that pathology – not sex – exists on a spectrum, one defined by severity.

DSD is no different from any other embryological pathology.^[5]^ No spectrum of orientations exists for the human heart, despite the numerous possible abnormalities (e.g. Tetralogy of Fallot). Rather, there is a spectrum of pathologies that can alter the heart’s ability to fulfil its purpose. The same is true of DSD. Suppose a person was born with a chimeric XX and XY genotype, and sufficient XY cells existed in one of the indifferent gonads for that gonad to become a testis. If that testis secreted sufficient testosterone in the crucial sexual differentiation window to orient the baby towards fatherhood and away from the default reproductive system’s path towards motherhood, that individual will be male – even if the other gonad has the XX genotype and became an ovary.

On the other hand, an XY individual with CAIS is a female whose body is organised around motherhood (notwithstanding that she would have non-functioning testicles in her abdomen). Embryologically, her body never received the androgen signal necessary to divert her away from the default female path.^[5,6]^ Just like the rocket previously mentioned, without the proper engine, her body still possesses and manifests a certain organisational character towards motherhood, even though her defunct gonads (undescended testes) are a male characteristic. Her DSD pathology has obstructed the organisational purpose of her reproductive system – i.e. functioning ovaries and uterus – but that purpose is still present. It is evident that lacking an ovary or uterus does not nullify someone’s femaleness; a cancer patient who has undergone hysterosalpingo-oophorectomy is still a woman. Although perhaps counter-intuitive, a person’s sex is not defined by gonads, since the absence or removal of gonads (a not uncommon procedure for females) does not annihilate one’s sex.

In teleological terms, ovaries or uteruses are accidental (albeit typical) female properties, and testicles are accidental male properties. Similar to the hysterosalpingo-oophorectomy case, a soldier whose testicles were destroyed in a bomb blast is still a male even though he can no longer reproduce. The bomb blast and cancer have hindered the reproductive systems from achieving their purpose but have not made the man ‘un-male’ and the woman ‘un-female’.

In human sports, aside from athletic prowess (and the possible addition of a specific weight), there are two fundamental entry requirements for participation: the participant must be human and belong to the sex in which they compete. It is vital that an individual’s eligibility to compete is only judged according to these categories. Platypuses do not participate in human swimming competitions because they are a different species, defined by different essential characteristics. However, a human swimmer with webbed feet, somewhat resembling those of a platypus, is no less human and more of a platypus because of them. Webbed feet are an accidental feature in humans, having no bearing on a human’s essential attributes, and thus should have no bearing on someone’s eligibility to compete as a human.

The same is true of sex. If someone is by essence a female, they should not be required to change their accidental features that are intrinsic to them as individuals to compete. There are only two reasonable alternatives, aside from eliminating sex as a category altogether. The first is to devise some complex algorithm to impose a handicap on performance based on genotypic and phenotypic traits. The second is for sports bodies to require corrective steps for every competitively advantageous trait, from webbed toes to gigantism. Yet there are no calls for male athletes with advantageous traits, like Michael Phelps or Gheorghe Mureșan, to undergo corrective procedures. Additionally, doing nothing is often a genuine medical option. Perhaps as a solution, every athlete with atypical attributes could be required to receive corrective treatment if that treatment is deemed safer than or as safe as no treatment, as it may be in Semenya’s case (despite the dearth of evidence on the trait’s advantageousness),^[3]^ but not in Gheorghe Mure’an’s case. However, these solutions seem infinitely complicated and only stand to make the process of determining ‘fair play’ hopelessly controversial.

Sport is not just about who trains harder, although tenacity and perseverance certainly play an integral role. Sporting prowess is also critically influenced by a set of genetic traits. How men and women capitalise on or overcome their inborn traits is a significant dynamic of high-performance athletics. In an era that ostensibly celebrates individual differences and fighting stereotypes, it would seem odd to punish athletes for possessing traits that, while arguably advantageous, have no essential bearing on their eligibility to compete. These authors submit that DSD athletes should be allowed to compete within the sex category to which they belong by essence, with whatever advantages or disadvantages they were born with.

## Conclusion

Since the CAF and IAAF judgments did not question Semenya’s sex, the proposal of these authors would ensure that she was treated with the same standard as other athletes with intrinsic advantages.
